# Hide and Go Seek: Finding the Locations of Ca, Zn, and S ions within LTA Zeolite Modified for H2S Capture

**DOI:** 10.1063/4.0000844

**Published:** 2025-10-27

**Authors:** Adeyemi D Ojaide, Miles Bradford, Stacey J Smith Doctorate, Roger G Harrison Doctorate

**Affiliations:** Brigham Young University, Provo, Utah, USA

## Abstract

Zeolite materials are porous crystalline structures that find widespread use in various applications, including catalysis and adsorption processes. Their effectiveness as adsorbents is significantly influenced by both the framework's ring structure and the species present within the channels. It is therefore crucial to understand both the framework's structure and the positioning of adsorbed species to optimize zeolites for adsorption applications. Our research aims to incorporate Zn(II) ions into Linde Type A (LTA) zeolites and assess their capacity for trapping sulfur from hydrogen sulfide (H_2_S). Elemental analyses indicate that our Zn-LTA has considerable potential for removing H_2_S from gas streams. To better understand this catalytic ability, the structure of the LTA framework and the locations of the adsorbed species were examined using X-ray diffraction (XRD). To date, most investigations into zeolite structure have primarily been theoretical and often lack supporting experimental evidence. Rietveld refinements of our XRD patterns from LTA, Zn-LTA, S-Zn-LTA, and regenerated Zn-LTA samples have, for the first time, provided experimentally derived locations for calcium, zinc, and sulfur ions within the LTA cages and channels.

